# Evaluation of dietary patterns and their impact on eye health among Saudi adults—A multi-regional cross-sectional analysis in Makkah, Riyadh, and Qassim

**DOI:** 10.3389/fnut.2024.1383725

**Published:** 2024-06-18

**Authors:** Ashokkumar Thirunavukkarasu, Bader Alanazi, Abdulrahman Alfaleh, Hani Hathath Alsulami, Sulaiman Abdullah Albudayr, Abdulrahman Saad Alotaibi, Reema Mohammed Alenezi, Araa Ghanem Alruwaili, Noor Oqalaa Alibrahim

**Affiliations:** ^1^Department of Family and Community Medicine, College of Medicine, Jouf University, Sakaka, Saudi Arabia; ^2^Department of Ophthalmology, College of Medicine, Jouf University, Sakaka, Saudi Arabia; ^3^Department of Nutrition, Northern Border University, Arar, Saudi Arabia; ^4^College of Medicine, Jouf University, Sakaka, Saudi Arabia

**Keywords:** nutrition, dietary pattern, Saudi adults, visual health, diet quality screener, lean protein intake

## Abstract

**Background and aim:**

Nutrition plays a vital role in maintaining and improving vision health. However, little is known about dietary intake habits and their correlation with vision health among adults in the Kingdom of Saudi Arabia (KSA). The present survey was aimed to assess dietary patterns and vision health among Saudi adults and to determine the association between dietary patterns and vision health.

**Methods:**

The present analytical study was carried out among 1,234 Saudi adults in the Makkah, Riyadh, and Qassim regions of KSA. We used the Arabic version of the National Eye Institute Visual Functioning Questionnaire-25 (NEI VFQ-25) and the diet quality screener (DQS). We applied Mann–Whitney U and Kruskal–Wallis tests to determine the association between vision function score and demographic characteristics. Furthermore, the Spearman correlation test was used to determine the relationship between the DQS and the NEI VFQ-25.

**Results:**

Of the studied population, the highest score obtained through the NEI VFQ-25 was in the social function domain (mean ± SD = 76.64 ± 18.63), followed by the general vision domain (mean ± SD = 75.21 ± 15.16) and was negatively correlated with age. Regarding dietary patterns, the intake of lean protein sources per week was the highest, with a mean intake of 4.17 days per week, followed by that of whole grains and milk or dairy products, with a mean intake of around four days per week. There was a significant correlation between various dietary intakes and visual function scores.

**Conclusion:**

The present survey underscores the significance of understanding regional dietary patterns and their implications for vision health. Furthermore, our study’s findings indicate a need for targeted nutritional intervention measures to improve the vision health of this population.

## 1 Introduction

Vision enables individuals to perform their daily tasks, such as learning, walking, shopping, and personal hygiene, without assistance from others (1, 2). Furthermore, as it affects all quality of life domains, visual problems are commonly associated with difficulties in physical function, emotional distress, and low socialization (personal, psychological, mobility, and social life) (3, 4). Visual impairment can cause substantial burden on the affected individual and the healthcare system (5, 6). The leading causes of visual impairment globally include diabetic retinopathy, cataracts, age-related macular degeneration, and glaucoma (7). The World Health Organization has launched VISION 2020: “The Right to Sight” to eradicate global blindness (8).

Dietary patterns have been linked with different aspects of vision health, including age-related macular degeneration, cataracts, glaucoma, and refractive errors. A nutritious eating regimen comprising fruits, vegetables, fatty fish, nuts, and various other food items has been recognized as advantageous for maintaining good eye health (9, 10). A diet that is rich in proteins and vegetables can reduce the risk of cataracts in middle-aged and elderly people (11). Similarly, a proper dietary regime for glaucoma patients is to maintain a normal weight, reduce excessive coffee intake, and enhance the intake of fruits and vegetables (12). Incorporating potent vitamins, antioxidants, and minerals into one’s dietary regimen can enhance vision and contribute to overall eye health. Numerous research findings highlight the potential benefits of lutein and zeaxanthin in mitigating the risk of chronic eye diseases (13, 14). Additionally, the significance of omega-3 fatty acids cannot be understated, as they are vital in supporting proper visual development and maintaining optimal vision health (15).

Traditionally, Saudi Arabia (KSA)’s dietary pattern consisted of dates, whole grains, and meat. However, in recent years, KSA has seen significant changes in lifestyle and nutritional patterns, primarily an increase in the intake of junk food, which is high in salt and high in cholesterol (16, 17). Hence, there is an increasing burden of non-communicable diseases (NCDs). Vision impairments have emerged as one of the most important public health challenges. In the majority of Eastern Mediterranean Region (EMR) countries, including KSA, blindness, and poor vision continue to be major public health concerns (18, 19). A study by Adam et al. (16) stated that the intake of sugar, meat, and animal fat has increased, and fruit and vegetable intake has changed among their study participants. Another study by Hammouh et al. (20) in 2023 revealed that most of their study participants had a higher proportion of low knowledge and poor dietary practices. A recent study by Mulpuri et al. (21) emphasized the importance of diet and vision health, and Francisco et al. (22) ascertained the significant assessment of dietary patterns to improve vision health and quality of life of millions of people and avoid high-cost vision surgeries.

Vision health is the main part of wellbeing, which is the basis of the quality of life and the capacity to do everyday tasks. Nutrition proves to be a key factor in vision health, and its specific dietary patterns are related to the prevention and management of different eye conditions. Hence, assessing the dietary patterns and their association with vision health in the KSA is critical to planning for the necessary dietary intervention programs tailored to the local context. Although the link between diet and vision health is well-known, studies on the relationship between these two factors among adults in the KSA are scarce. Furthermore, dietary patterns are constantly changing among the population. Hence, continuous assessment of the dietary patterns in different cultural settings is essential. Therefore, this research aimed to conduct a nutrition and vision health survey among adults in three regions (Makkah, Riyadh, and Qassim) of Saudi Arabia to assess their dietary patterns and their correlation with vision health.

## 2 Participants and methods

### 2.1 Study description

A quantitative cross-sectional study was conducted among adults aged 18 years and older from May to October 2023 in three regions of Saudi Arabia–Qassim, Makkah, and Riyadh. Participants were recruited from public places such as malls, parks, and local community centers in Qassim, Makkah, and Riyadh. We chose this age group to focus on adults who are more likely to make independent dietary choices and experience age-related vision changes. We excluded patients who were diagnosed with chronic diseases, such as diabetes, hypertension, adults with already existing eye problems or injuries, and those aged above 65 years.

### 2.2 Sample size estimation and sampling method

Considering the limited studies available in this context, we have taken 50% as the expected proportion (p) to estimate the sample size. Numerous authors use this conservative method if the limited study is available to estimate the p in Cochran’s sample size estimation formula to get the largest size and sufficient power to detect significant associations. We used the WHO sample size calculator that uses the same principles of Cochran’s formula (*n* = z^2^pq/e^2^) with a 95% confidence interval and 5% margin of error. The total sample estimated size was 384, and we rounded it to 400. Considering three regions, the research team decided to recruit a minimum of 400 participants from each region (Total = 1,200). The research team applied a convenience sampling method to recruit the participants. In this method, the data collectors made a stall in public places and invited them to participate.

### 2.3 Data collection procedure

We obtained ethical clearance from the regional research ethics committee, general directorate of health affairs, Gassim region, KSA (Approval number: 607/44/14429, Dated: 03.05.2023). The data collectors invited the participants from public places, as mentioned earlier. Interested individuals were given information about the study and invited to participate. All participants were required to give informed consent before participating in the study. The survey was administered through a Google form link on the data collectors’ personal devices to participate in the survey. The survey was administered in the Arabic language. The data were collected anonymously, and the responses were accessed only by the principal investigator to protect the data. The data collection tool for this study was a self-administered survey that consisted of three sections: demographic information, the National Eye Institute Visual Functioning Questionnaire-25 (NEI VFQ-25), and the Diet quality assessment questionnaire. In the first section of the Google form, the participants filled their background characteristics such as age, gender, occupation, marital status, and level of education of the participants.

NEI VFQ-25 (second section) consists of 25 questions rated on a Likert scale wherein patients were asked to score the degree of difficulty associated with specific visual symptoms or tasks, including reading newspapers or driving (23). The questions were grouped into the following subdomains: general health, general vision, ocular pain, near vision activities, distance vision activities, and social functioning. In the scope of each subdomain, the participants gave answers that showed their individual experiences and views on the issues related to vision. In the domain of general health, the participants rated their overall health in relation to their vision problems, with scores ranging from 0 to 100, and the higher the score, the better the health. For the general vision subdomain, the participants again evaluated the vision aspects, such as vision clarity and satisfaction, on a scale from 0 to 100. Ocular pain was measured by the subjects who reported how often and how severe the pain or discomfort was due to the vision problems on a Likert scale. The near vision activities were assessed by the participants who specified the level of difficulty they had in tasks like reading or using electronic devices, from “No difficulty” to “Unable to do,” using a Likert scale. Besides, participants also rated the ability to do distance vision activities like driving or watching television. Finally, the social functioning of the participants was evaluated by the rating of their social interactions and participation in leisure activities despite vision problems. Responses were noted on Likert scales or numerical rating scales; thus, the researchers knew the participants’ thoughts on the vision-related quality of life in different domains. According to the NEI VFQ-25 scoring system, the subdomains were assessed and converted to a scale ranging from 0 to 100, where a score of 100 indicated the highest level of function. The composite (overall) score for the NEI VFQ-25 was derived as the mean of all subscale scores except the general health score. The NEI VFQ-25 has been used in various settings and is proven valid and reliable (24–26). Its extensive use in research makes it a suitable choice for capturing the subjective experiences and functional abilities related to vision health among our study participants. In the third section, diet quality was assessed by a dietary quality screener (DQS) consisting of questions regarding the diet intake per typical week, which included frequency of intake of fruit, vegetables, whole grains, and lean protein sources, variety of types of vegetables, and adequacy of milk and dairy product consumption. Participants indicated how often they consumed these items, with response options ranging from “Less than once a week or never” to “Daily or almost daily.” The questionnaire was prepared for dietary quality screening from various dietary questionnaires according to the study population through a focused group discussion by the family medicine, public health, and nutrition experts based on previous works of literature (27–29). Hence, these two tools provided a quick and reliable method for examining the relationship between diet and vision health outcomes. The questionnaire was then translated into Arabic language and re-translated to check the appropriateness of the questions. As mentioned earlier, the prepared data collection form consisted of three sections and was initially tested through a pilot study among 30 eligible participants. All pilot study participants have given feedback that the instrument is clear and easy to understand. On average, it took 10 min to complete the questionnaire by the pilot study participants. The coefficient alpha (Cα) value obtained for the data collection tool is above 0.70 for both NEI VFQ-25 (Cα = 0.77) and DQS (Cα = 0.82) The pilot study participants’ data were included in the overall analysis.

### 2.4 Statistical analysis

We analyzed the collected data statistically using IBM SPSS version 21 software. The demographic variables are expressed in frequencies and proportions. The dietary scores and visual function scores were expressed in mean and standard deviation. We applied Mann–Whitney U and Kruskal–Wallis tests to determine the association between vision function score and demographic characteristics. Furthermore, the Spearman correlation test was used to determine the relationship between DQS and NEI VFQ-25 variables. A *p*-value less than 0.05 derived from a two-tailed test was set as statistically significant.

## 3 Results

A total of 1,234 participants were included in the study, of which 403 were recruited from Qassim, 417 from Riyadh, and 414 from Makkah. The age of the participants was almost similar in all three regions, with a mean of 35.4 years. The occupation of the participants had almost the same distribution in the three regions with a higher proportion of self-employed and private sector working people except in Makkah, where the number of people working in the government sector was high, followed by private sector workers and self-employed. Most of the participants were married, followed by single, with less than 15% constituting divorced and widowed. Almost 80% of the study population in all the regions had either a bachelor’s degree or diploma, with 6 to 8% having postgraduate degrees. Almost 10% had completed secondary grade of school education (Table 1).

**TABLE 1:** Demographic characteristics of the study population (*n* = 1,234).

Demographic characteristics		Region			Total (*n* = 1,234)
		Qassim (*n* = 403)	Riyadh (*n* = 417)	Makkah (*n* = 414)	
Age (years) (mean ± SD)		34.2 (± 9.2)	34.9 (± 9.1)	37.1 (± 11.1)	35.4 (± 9.9)
Gender	Male	255 (63.3%)	277 (66.4%)	292 (70.5%)	824 (66.8%)
	Female	148 (36.7%)	140 (33.6%)	122 (29.5%)	410 (33.2%)
Occupation	Private sector	113 (28%)	136 (32.6%)	105 (25.4%)	354 (28.7%)
	Unemployed	36 (8.9%)	28 (6.7%)	33 (8%)	97 (7.9%)
	Government sector	76 (18.9%)	72 (17.3%)	122 (29.5%)	270 (21.9%)
	Students	50 (12.4%)	44 (10.6%)	62 (15%)	156 (12.6%)
	Retired	9 (2.2%)	8 (1.9%)	28 (6.8%)	45 (3.6%)
	Self-employed/business	119 (29.5%)	129 (30.9%)	64 (15.5%)	312 (25.3%)
Marital status	Single	163 (40.4%)	165 (39.6%)	149 (36%)	477 (38.7%)
	Married	179 (44.4%)	188 (45.1%)	232 (56%)	599 (48.5%)
	Divorced	52 (12.9%)	52 (12.5%)	29 (7%)	133 (10.8%)
	Widow	9 (2.2%)	12 (2.9%)	4 (1%)	25 (2%)
Education status	Diploma	164 (40.7%)	172 (41.2%)	96 (23.2%)	432 (35%)
	Bachelor’s degree	173 (42.9%)	181 (43.4%)	228 (55.1%)	582 (47.2%)
	Secondary	42 (10.4%)	34 (8.2%)	56 (13.5%)	132 (10.7%)
	Postgraduate	24 (6.0%)	30 (7.2%)	34 (8.2%)	88 (7.1%)
Income (SAR) (1 USD = 3.75 SAR)	< 5,000	67 (16.6%)	44 (10.5%)	53 (12.8%)	164 (13.3%)
	5,000 to 7,000	137 (34.0%)	163 (39.1%)	194 (46.9%)	494 (40.0%)
	> 7,000	199 (49.4%)	210 (50.4%)	167 (40.3%)	576 (46.7%)

Regarding the visual functioning assessment, the mean general vision score and other vision scores were almost 75, except for general health, which was 65.3. The mean overall vision score was 74.43 (±15.29) and had a negative correlation with age. Near vision activity and general vision had a high negative correlation with increasing age, followed by distant vision activity and social functioning score. The correlation of age with the scores was statistically significant (Table 2).

**TABLE 2:** Visual functioning scores and their correlation with age (*n* = 1,234).

NEI VFQ-25 scores	Mean (± SD)	Correlation with age
General health	65.3 (± 21.35)	-0.127**
General vision score	75.21 (± 15.16)	-0.223**
Ocular pain score	70.46 (± 38.11)	0.068*
Near vision activities score	74.68 (± 17.02)	-0.317**
Distance vision activities score	75.2 (± 16.58)	-0.172**
Social functioning score	76.64 (± 18.63)	-0.173**
Overall visual function score	74.43 (± 15.29)	-0.228**

*Correlation is significant at 0.05 level;

**Correlation is significant at 0.01 level.

All the demographic variables were significantly related to the visual function score. Males had a better visual function than females. Government sector employees had a higher visual function, followed by students, those in the private sector, and unemployed individuals. The visual function scores were low among retired people and self-employed. Regarding marital status, single and married people had better visual function than divorced and widowed people. People residing in Makkah had higher visual function scores, followed by Qassim and Riyadh (Table 3).

**TABLE 3:** Association between visual function score and demographic characteristics.

Variables		Overall Visual function score	Kruskal–Wallis *p*-value
Gender	Male	75.9 (± 15.5)	0.001^#^
	Female	71.5 (± 14.5)	
Occupation	Private sector	72.5 (± 13.3)	0.001
	Unemployed	73.5 (± 15.8)	
	Government sector	80.6 (± 17.8)	
	Students	79.8 (± 13.8)	
	Retired	68.9 (± 20.2)	
	Self-employed/business	69.7 (± 12)	
Marital status	Single	76.6 (± 14.4)	0.001
	Married	74.6 (± 15.7)	
	Divorced	69.6 (± 13.4)	
	Widow	56.2 (± 13)	
Education status	Diploma	71.3 (± 12.8)	0.001
	Bachelor	75.1 (± 16)	
	Secondary	79.3 (± 15.7)	
	Postgraduate	78.5 (± 18)	
Region	Qassim	74.4 (± 15.1)	0.001
	Riyadh	71.5 (± 13.3)	
	Makkah	77.5 (± 16.7)	
Income (SAR)	< 5,000	72.4 (± 13.8)	0.019
	5,000 to 7,000	74.8 (± 14.3)	
	> 7,000	75.6 (± 15.4)	

^#^*p*-value by Mann–Whitney U test.

Lean protein sources’ weekly intake was the highest, with a mean intake of 4.17 days per week, followed by whole grains and milk or dairy products, with a mean intake of around four days per week. Mean vegetable intake, dark green vegetable intake, and orange vegetable intake were more than three days per week, and fruit intake was less than three days per week (Table 4).

**TABLE 4:** Dietary quality screener assessment results (*n* = 1,234).

Dietary quality screener	Less than once a week or never	1 to 2 times a week	3 to 4 times a week	5 to 6 times a week	Daily or almost daily	Days per week mean (± SD)
Fruits intake	127 (10.3%)	482 (39.1%)	345 (28%)	194 (15.7%)	86 (7%)	2.92 (± 1.99)
Vegetable intake	41 (3.3%)	361 (29.3%)	444 (36%)	261 (21.2%)	127 (10.3%)	3.58 (± 1.92)
Whole grains intake	60 (4.9%)	217 (17.6%)	402 (32.6%)	427 (34.6%)	128 (10.4%)	4.03 (± 1.92)
Lean protein sources intake	39 (3.2%)	184 (14.9%)	451 (36.5%)	419 (34%)	141 (11.4%)	4.17 (± 1.82)
Milk or dairy products intake	45 (3.6%)	248 (20.1%)	425 (34.4%)	357 (28.9%)	159 (12.9%)	4.01 (± 1.93)
Dark green vegetables intake	66 (5.3%)	315 (25.5%)	449 (36.4%)	285 (23.1%)	119 (9.6%)	3.6 (± 1.94)
Orange vegetable intake	40 (3.2%)	311 (25.2%)	437 (35.4%)	309 (25%)	137 (11.1%)	3.77 (± 1.92)

There was a significant correlation between various dietary intake scores and visual function scores. Vegetable intake, dark green vegetable intake, orange vegetable intake, lean protein sources intake, and milk or dairy products intake significantly correlated with general health, general vision, near vision, distant vision, and social function scores. Fruit intake is positively correlated with general health, distant vision, and social functioning, whereas whole grain intake is positively correlated with general vision, near vision, and distant vision scores (Table 5).

**TABLE 5:** Correlation between dietary intake and visual functions.

	Fruits intake (days/week)	Vegetable intake (days/week)	Whole grains intake (days/week)	Lean protein sources intake (days/week)	Milk or dairy products intake (days/week)	Dark green vegetables intake (days/week)	Orange vegetables intake (days/week)
General health	0.114**	0.138**	-0.030	0.104**	0.070*	0.109**	0.171**
General vision score	-0.004	0.295**	0.126**	0.243**	0.235**	0.116**	0.109**
Ocular pain score	0.021	-0.038	-0.048	-0.057*	-0.024	0.083**	0.060*
Near vision activities score	0.002	0.266**	0.117**	0.234**	0.195**	0.157**	0.141**
Distance vision activities score	0.038	0.287**	0.075**	0.202**	0.202**	0.184**	0.165**
Social functioning score	0.126**	0.234**	0.009	0.221**	0.162**	0.232**	0.233**

*Correlation is significant at 0.05 level;

**Correlation is significant at 0.01 level.

## 4 Discussion

This study aimed to address the gap in understanding the relationship between dietary habits and vision health among Saudi adults, providing valuable insights for public health interventions and strategies to preserve and enhance vision. The current study reported the visual functions among the population and showed that the mean overall vision score was 74.43. Population-based studies showed that the mean visual function score varies from 83 to 93 across the age groups, which signifies that the current study population had a lower visual function score than previous studies (24, 30). The variations across the studies could be attributed to the dietary patterns, lifestyles followed in the Arabian region and variations in data collection tools in the studies as mentioned above.

The age was negatively correlated with the visual function scores, which were statistically significant. Other studies have investigated the impact of age-related declines in visual function on physical health. One study revealed that visual impairment is associated with poor physical health, including physical limitations, difficulty with activities of daily living, and reduced quality of life (31). However, community-based factors could alleviate the effects of vision loss on physical outcomes, highlighting the importance of public health endeavors to address visual impairment in older adults (31). One study revealed that decreases in visual function associated with aging significantly affect the health and overall wellbeing of elderly individuals. These declines occur across various levels of sensory and perceptual processing (32).

The visual function scores showed differences for all the demographic variables. Men had better visual function than women. This is supported by the study by Khandekar and Mohammed et al. (33), which showed that the age-adjusted prevalence of blindness in women was 3% higher than that in men. The current study showed that widow and divorced people had low visual function, which is supported by the study by Ezeh et al. (34) which had similar results. The visual function score was low among retired people and self-employed people, which may be attributed more to their age correlation. Region-wide differences in the visual functions may be attributed to their diet and lifestyle practices.

The current study analyzed the dietary patterns followed by the people in three regions of KSA. It was observed that people had a mean of more than four days per week of consumption of lean protein sources, whole grains, and dairy products, followed by a mean intake of vegetables for more than three days per week, followed by fruits, indicating the consumption of a mixed diet with a good source of carotenoids, vitamins, and minerals along with polyunsaturated fatty acids. Reasonable evidence suggests that dark-green leafy vegetables, significantly those high in lutein and zeaxanthin, may help prevent the onset and progression of age-related macular degeneration (AMD) and help lower the risk of some subtypes of cataracts and slow the advancement of glaucoma and diabetic retinopathy (35). Observational studies have revealed that long-chain omega-3 fatty acids, primarily derived from fish, are protective against certain kinds of visual impairment, specifically AMD (15, 36, 37).

The significant correlation between various dietary intakes and visual function scores in the present study further supports this finding. Vegetable intake, especially dark green vegetables and orange vegetables was significantly correlated with general health, general vision, near vision, distant vision, and social function scores. Similarly, the Eye Disease Case-Control Study (EDCC) found that increased intake of dark-green leafy vegetables was associated with a reduced risk of neovascular AMD (OR–0.57) (38). The Age-Related Eye Disease Study 2 (AREDS2) trial examined lutein and zeaxanthin (LZ) supplementation and revealed that sufficient dietary intake of LZ may protect against AMD progression (13). Fruits intake had a positive correlation with general health, distant vision, and social functioning. This is being reflected by many studies. Cross-sectional studies in urban India found a significant association of diabetic retinopathy with low dietary fiber intake in patients with type 2 diabetes (OR–2.24) (39). Blue Mountains Eye research explored that increased consumption of combined vitamins C, E, zinc, and beta-carotene reduces the chances of developing cataracts (OR–0.51) (40). A higher intake of fruits and increased vegetable intake reduced glaucoma risk among the population, as stated by several authors (12, 41).

Lean protein source intake and milk or dairy product intake significantly correlated with general health, general vision, near vision, distant vision, and social function score. In contrast, whole grain intake had a positive association with general vision, near vision, and distant vision scores. These correlations are evidenced in many studies. Cohort studies have demonstrated that increased fish consumption is negatively associated with neovascular AMD development (OR–0.25) (40), (RR 0.65) (42). A meta-analysis by Chong et al. (43) has demonstrated that eating more than two servings of fish per week decreases the incidence of both early and late AMD, as does consuming high levels of omega-3 fatty acids (OR–0.62). Merle et al. (44) reported that higher dietary intake of vitamin D intake was associated with a lower risk of developing AMD. Reduced consumption of milk products and calcium was significantly linked with adverse retinal vascular symptoms (45, 46).

Oxidative stress and inflammation are believed to be essential in the pathogenesis of AMD, diabetic retinopathy, cataracts, and glaucoma are interconnected, implying that dietary interventions aimed at reducing these conditions could potentially enhance the outcomes of all four disorders simultaneously. Dietary intake of certain foods to decrease these stresses has been well-documented in AMD, diabetic retinopathy (DR), and cataracts (47). Visual impairment has important repercussions for both visually impaired individuals and on the health system. Changes in diet and lifestyle, such as reducing the risk of hyperglycaemia and eating more dark-green leafy vegetables, have the potential to delay or stop the onset of disease and have positive effects on preventing the development of other systemic diseases. Given the current burden of visual impairment on individuals and healthcare systems, further research is needed to determine whether implementing proper dietary and lifestyle measures can effectively reduce the risk of visual impairment.

The survey team performed this vision and nutrition survey using a standard and validated questionnaire. However, we suggest that the readers consider the following constraints while reading the present nutrition survey findings. Firstly, we included only three regions of the KSA. However, the dietary patterns may vary in other regions as sociocultural norms are different in several parts of the KSA. Secondly, we used a convenience sampling method, and the limitations of this method need to be considered. Finally, this nutrition and vision survey used a cross-sectional approach. Hence, the causal association between dietary patterns and vision health cannot be established, as this can be done only through prospective studies.

## 5 Conclusion

The visual function scores were low among the adults in the Qassim, Makkah, and Riyadh regions of Saudi Arabia. The visual functions were low among women, widowed and divorced people, retired people, self-employed people, and Riyadh residents. The mean days of consumption of lean protein sources, whole grains, and dairy products, followed by a mean intake of vegetables, were between three and four days per week, indicating the participants’ poor dietary patterns. We found a significant positive correlation between the NEI VFQ-25 score and fruit and vegetable intake. Hence, there is a need for dietary advice that can be provided through health education, health promotion, and community participation to prevent visual impairment and avoidable blindness. Finally, further exploratory prospective studies are warranted to identify the temporal association between vision health and dietary patterns.

## Data availability statement

The raw data supporting the conclusions of this article will be made available by the authors, without undue reservation.

## Ethics statement

The studies involving humans were approved by the Institutional Review Board, General Directorate of Health Affairs, Al-Gassem Region, Saudi Arabia. The studies were conducted in accordance with the local legislation and institutional requirements. The participants provided their written informed consent to participate in this study.

## Author contributions

AT: Conceptualization, Data curation, Formal analysis, Funding acquisition, Methodology, Resources, Supervision, Writing – original draft. BA: Conceptualization, Methodology, Resources, Supervision, Visualization, Writing – original draft. AAlf: Conceptualization, Methodology, Project administration, Software, Validation, Visualization, Writing – original draft. HA: Conceptualization, Data curation, Formal analysis, Resources, Validation, Visualization, Writing – original draft. SA: Data curation, Formal analysis, Methodology, Project administration, Software, Validation, Writing – original draft. AAlo: Conceptualization, Data curation, Formal analysis, Methodology, Project administration, Writing – original draft. RA: Data curation, Formal analysis, Methodology, Project administration, Software, Validation, Visualization, Writing – review & editing. AAlr: Conceptualization, Data curation, Investigation, Software, Validation, Writing – review & editing. NA: Conceptualization, Data curation, Validation, Writing – review & editing.
